# Influence of degree of learning on rate of forgetting of tonal sequences

**DOI:** 10.3758/s13421-024-01597-6

**Published:** 2024-07-17

**Authors:** Karim Rivera-Lares, Alan Baddeley, Sergio Della Sala

**Affiliations:** 1 Human Cognitive Neuroscience, Psychology Department, University of Edinburgh, Edinburgh, UK; 2 Department of Psychology, University of York, Heslington, York UK

**Keywords:** Forgetting rates, Nonverbal material, Initial degrees of learning

## Abstract

Initial performance is frequently equated in studies that compare forgetting rates across groups. However, since the encoding capacity of different groups can be different, some procedures to match initial degree of learning need to be implemented, adding confounding variables such as longer exposures to the material, which would create memories of a different age. Slamecka and McElree *Journal of Experimental Psychology: Learning, Memory, and Cognition*, *9*, 384–397, (1983) and our previous work found that the rate of forgetting was independent from initial degree of learning using verbal material. The present study seeks to determine whether this pattern holds true when undertaken with nonverbal material. In two experiments, we manipulate initial degree of learning by varying the number of presentations of the material and studying the effect on the forgetting rates. A set of 30 tonal sequences were presented to young, healthy participants either once or three times. Forgetting was evaluated in a yes/no recognition paradigm immediately and 1 hour or 24 hours after the study phase. A different subset of 10 sequences was tested along with 10 nontargets at each retention interval. The results of these experiments showed that initial acquisition was modulated by the number of repetitions. However, the forgetting rates were independent of initial degree of learning. These results are in keeping with the pattern found by Slamecka and McElree, and in our own previous studies. They suggest that the pattern of parallel forgetting after different levels of initial learning is not limited to verbal material.

**Supplementary Information:**

The online version contains supplementary material available at 10.3758/s13421-024-01597-6.

The study of forgetting has, in recent years, been relatively neglected, despite its relevance for theory and day-to-day life. One important question in the study of forgetting is whether initial degree of learning has any influence on forgetting rates. This question is relevant due to its theoretical implications and also to inform the methodologies used in studies that seek to compare long-term forgetting between groups, such as younger and older adults, or healthy controls and clinical populations. If researchers assume that initial degree of learning influences long-term forgetting, they will match initial acquisition for both groups via different procedures such as more or longer exposures to the material (e.g., Gaudino et al., 2001; Huppert & Piercy, 1978; Simone et al., 2013; Stamate et al., 2020; Walsh et al., 2015). However, exposing groups to a different number of repetitions or to longer presentations of the stimuli implies that the memories tested are of a different age, which, according to Jost’s (1897) second law of forgetting, are of different strengths. The present study aims to investigate whether the rate of forgetting is independent from initial degree of learning.

There is a critical problem in comparing the rate of forgetting found in two groups that start at different levels of initial performance: Should forgetting be measured in percentage terms, as proposed by Loftus (1985a, b), in which case the loss of the same number of items will be greater for the lower performing participants, or in terms of number of items lost, as proposed by Slamecka and McElree (1983)? As pointed out by Loftus (1985a) answering this question will depend on one’s theory of forgetting, with different theorists proposing different forgetting functions (Fisher & Radvansky, 2019; Radvansky, Doolen et al., 2022a, Radvansky, Parra et al., 2022b; White, 2001; Wixted, 2022; Yang et al., 2016). A problem with using proportion of losses as a measure of forgetting rates instead of absolute numbers is that it introduces an arbitrary dependence on the intercept levels (Slamecka & McElree, 1983). Thus, we decided against measuring forgetting as a proportion of items lost. We use the absolute number of items forgotten instead. It is important to note that using proportions instead of absolute numbers would produce different results. Imagine two different groups exposed to 10 items. Group A learns eight items initially, whilst Group B learns four. If both groups forget two items in a subsequent test, that will represent a loss of 25% for Group A and of 50% for Group B. This would be interpreted as Group B forgetting more than Group A, when both groups are forgetting at the same rate.

Slamecka and McElree (1983) investigated the relationship between two levels of initial learning and their subsequent forgetting rates. The different degrees of initial learning were achieved by exposing participants to a different number of repetitions of the material. Forgetting was measured using different types of tests at three retention intervals (30 s, 3 days, and 5 days). In all experiments, more repetitions resulted in a higher degree of initial acquisition, and longer delays resulted in lower performance. However, there was no interaction between these variables, indicating that the rates of forgetting are not modulated by initial degree of learning, which suggests that their study has been overlooked in the field (e.g., Derwinger et al., 2005; MacDonald et al., 2006; Nelson et al., 2016; Zerr et al., 2018).

One of the concerns of our laboratory is to investigate under which conditions parallel forgetting occurs and persists. In our previous work (Rivera-Lares et al., 2022, 2023), we used verbal material to investigate this matter, and found that the forgetting curves were indeed independent from the initial level of acquisition, as postulated by Slamecka and McElree (1983). If the same pattern appears consistently with a variety of materials and types of tests, we could be confident that a general empirical principle of forgetting has been established (Wixted, 1990).

A study by Sense et al. (2016) found that different types of material produced different rates of forgetting. Most long-term forgetting studies have used verbal materials such as words, sentences, and prose and nonwords, or visual material such as landscapes and photographs (see the review in Roediger et al., 2010). To assess the reliability of the pattern of parallel forgetting curves starting from different initial degrees of learning, it is important to investigate whether this pattern is unique to verbal material or if it extends to nonverbal material as well. Therefore, in this study, we used auditory nonverbal material, which has the particularity of being more difficult to rehearse than verbal material.

Stalinski and Schellenberg (2013) explored retention of music clips with different degrees of likeability and found that participants remembered better the excerpts they liked more. However, this study focused on immediate retention, assessing participants’ memory only once after exposure, rather than repeatedly testing participants in order to explore forgetting rates. Kauffman and Carlsen (1989) assessed three groups of participants according to their musical knowledge: experts, novices, and non-musicians. They were asked to listen to pairs of music excerpts, which were tested at delays from immediate to 180 seconds, using recognition. A significant difference in initial degree of learning was found between the experts and the nonmusicians, but their rate of forgetting was comparable.

To achieve different initial levels of performance, we ran two experiments exposing participants to either one or two repetitions of a list of 30 audio clips, and then tested how many of these clips they could correctly recognize at three retention intervals. To minimize testing effects, we tested a different subset of both previously presented and novel audio clips at each of the three retention intervals.

## Experiment 1

Participants were exposed to either one or two repetitions of a list of novel music excerpts and tested using a yes/no recognition paradigm at three retention intervals. At each interval, a different subset of the old music clips was tested along with the same number of new clips. Number of repetitions was between subjects, and retention interval tests were within-subjects.

### Method

#### Participants

Sixty students from the University of Edinburgh (*M*_age_ = 21.28 years, *SD =* 2.41, range: 18–28; 25 men), were recruited through the University student experimental panel. All participants provided written, informed consent before starting the experiment, and were given credits for their time upon completion. All had normal or corrected-to-normal vision and normal hearing. This study was approved by the School of Philosophy Psychology and Language Sciences Research Ethics Committee, at the University of Edinburgh.

The participants were randomly allocated: 30 (*M*_age_ = 21.47 years, *SD =* 2.30, range: 18–28; 13 men) to the one repetition condition and 30 (*M*_age_ = 21.10 years, *SD* = 2.65, range: 18–28; 12 men) to the two repetitions condition. There was no significant difference in age between the one repetition and the two repetition groups, *t*(56.84) = 0.58, *p* = .56.

#### Materials

Sixty-five-second music clips were used for this experiment. All of them were taken from short melodic phrases played on the piano in a variety of moods and styles. Half of them were taken from the YouTube audio library, and to ensure that participants had not heard them before, we used only music labelled as new on the website. At the time of the recording, there were not sufficient clips on the YouTube audio library, so additional audio clips were specifically composed for this experiment and digitally recorded. An attempt was made for the composed clips to not stand out from the material taken from the YouTube library.

Thirty of these clips were randomly selected to be presented during the study phase (hereafter, targets) as material to-be-remembered. The remaining 30 were used as nontargets, along with the 30 targets during the testing phase. The rate of presentation was of five seconds per clip, with a two-second interval between each clip.

#### Procedure

All participants were tested in person in a quiet room at the University of Edinburgh. The experiment consisted of a study phase and a test phase. During the study phase, the experimenter asked the participant to listen carefully to the music clips that were to be played from a computer and stated whether the clips were going to be presented once or twice. The participants were aware that the experiment was run in three sessions, with the second and third involving a memory task. A study trial consisted of the list of 30 targets presented in a different random order for each participant. If the participant was in the two repetition condition, the first 30 targets were randomized for the first presentation and then randomized again for the second.

The test phase consisted of a yes/no recognition test, which took place immediately, 1 hr, and 24 hr after the end of the study phase. All participants were tested at each delay using a different sample of 10 targets and 10 nontargets. Participants were asked to listen to the whole 5-s music clip before offering a response and to guess if they did not remember. Only when the participant had responded could they hear the next item. The instruction was to say “yes” to indicate an old item. previously heard during the study phase, or “no” to indicate a new item. The responses were recorded manually.

#### Planned analyses

Forgetting studies usually employ general linear models (e.g., repeated-measures analysis of variance [ANOVA]), which require the averaging of individual measurements, assuming that the rate of forgetting across individuals is the same, and that individual deviation from the mean reflects error. In contrast, mixed-effects models are superior for the analysis of forgetting data, as they allow for varying initial degrees of learning (called random intercepts in the model) and varying individual forgetting slopes (called random slopes).

We present the hit rates and false-alarm rates in order to show a clear picture of changes in memory over time, since the rate of hits captures the amount of information accessible in memory at each point in time (Radvansky, Doolen et al., 2022a). Two generalized linear mixed-effects models were fitted, one for the analysis of the hit rates (rates of targets identified as such), and another one for the analysis of the false-alarm rates (rates of nontargets incorrectly identified as targets). In both models, the dependent variable was binary: hit (1) or no hit (0); false alarm (1) or no false alarm (0). Number of repetition and retention interval were the independent variables (called fixed effects in the model). To account for the different learning capacities of the participants and the different difficulties of the items, random intercepts over participants and items were included, as well as a random slope for retention interval over participants allowing the rate of forgetting to vary per participant. The models were fitted using a Bernoulli distribution. All the analyses were carried out using Bayesian inference, an alternative to the more well-known Frequentist framework. Bayesian inference has been found to produce more accurate predictions (Kruschke et al., 2012), its results are intuitively plausible and their interpretation is straightforward. All the models were fitted using the Stan modelling language (Carpenter et al., 2017) and the R package *brms* (Bürkner, 2017, 2018) using the default priors. Parameter uncertainty is described by the 95% credible interval (CI) of the posterior distribution in addition to the mean parameter value. Substantial evidence of an effect is said to be found when the CI does not contain zero. The generalised linear mixed-effects models used in this work perform logistic regressions, thus, the estimates are given on the log odds ratio scale, so the value of the estimate reflects the change in the probabilities of correctly recalling an item in log odds ratio (Christensen, 2006).

The performance of four participants from the one repetition condition, and three from the two repetition condition were excluded from the final analyses as they correctly identified less than the number of items that can be identified purely by chance, which was interpreted as lack of engagement with the task. The hit rates and false-alarm rates were analyzed independently.

### Results

**Fig. 1:**
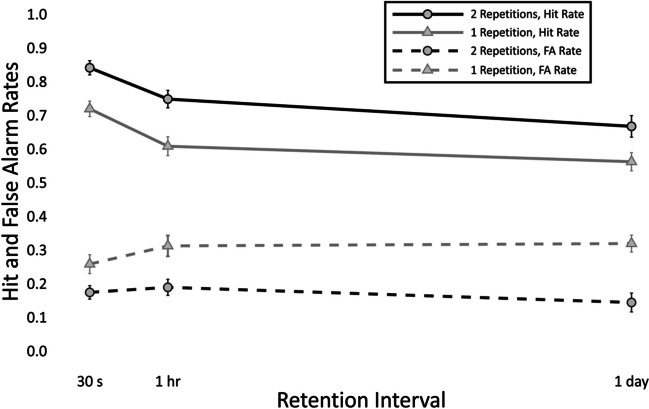
Means and standard errors of hit rates and false-alarm rates at each combination of number of repetitions and retention interval

The means and standard errors of hit rates and false-alarm rates are depicted in Fig. 1. The values are reported in the Supplementary Material.

#### Hit rates

There was substantial evidence for an effect of repetition at immediate recall (*b =* −0.73, *SD =* 0.23, CI [−1.19, −0.29]). There was substantial evidence for an effect of delay from immediate to 1 hr (*b =* −0.58, *SD =* 0.22, CI [−1.02, −0.14]), immediate to 24 hr (*b =* −0.98, *SD =* 0.22, CI [−1.42, −0.55]), and 1 hr to 24 hr (*b =* −0.41, *SD =* 0.2, CI [−0.8, −0.02]). There was no evidence of an interaction between delay and repetition from immediate to 1 hr (*b =* 0.06, *SD =* 0.3, CI [−0.53, 0.64]), immediate to 24 hr (*b =* 0.27, *SD =* 0.29, CI [−0.31, 0.84]), or 1 hr to 24 hr (*b =* 0.21, *SD =* 0.27, CI [−0.33, 0.75]).^1^

#### False-alarm rates

There was substantial evidence of an effect repetition at immediate recall (*b =* 0.53, *SD =* 0.25, CI [0.04, 1.02]). There was no evidence of an effect of delay from immediate to 1 hr (*b =* 0.09, *SD =* 0.24, CI [−0.37, 0.54]), immediate to 24 hr (*b =* −0.26, *SD =* 0.25, CI [−0.75, 0.23]), or 1 hr to 24 hr (*b =* −0.35, *SD =* 0.25, CI [−0.84, 0.14]). There was no evidence of an interaction between delay and repetition from immediate to 1 hr (*b =* 0.17, *SD =* 0.31, CI [−0.44, 0.79]), immediate to 24 hr (*b =* 0.57, *SD =* 0.32, CI [−0.07, 1.2]), or 1 hr to 24 hr (*b =* 0.39, *SD =* 0.32, CI [−0.23, 1.02]).

### Discussion

Initial degree of learning was successfully manipulated through varying the number of repetitions. More repetitions produced a higher initial degree of learning. There was an effect of delay, with each subsequent test reducing the hit rates. The difference in the hit rates at the immediate test remained constant across retentions. This lack of interaction suggests that rate of forgetting is independent of initial degree of learning. The false-alarm rates were only affected by the number of repetitions, with more repetitions reducing the rate of false alarms. No effect of retention interval was found, and no interaction between delay and degree of learning.

Most of the forgetting occurred in the first interval, in line with the Ebbinghaus forgetting curve (1885/1964). Our results are consistent with studies which found no differences in forgetting rates after different levels of acquisition using verbal material (e.g., Rivera-Lares et al., 2022, 2023; Slamecka & McElree, 1983).

A replication of the above findings was run as Experiment 2. Due to the COVID-19 pandemic, Experiment 2 was carried out online.

## Experiment 2

Experiment 2 was similar to Experiment 1, except that it was carried out online.

### Method

#### Participants

Sixty students from the University of Edinburgh (*M*_age_ = 20.40 years, *SD* = 2.34, range: 18–26; 12 men) were recruited through the University’s student experimental panel and the job portal. All participants provided written, informed consent before starting the experiment and were given credits or payment for their time upon completion. All reported normal hearing and normal or corrected-to-normal vision.

The participants were randomly allocated: 30 (*M*_age_ = 21.12 years, *SD* = 2.21, six men) to the one repetition condition and 30 (*M*_age_ = 20.70 years, *SD* = 2.44, six men) to the two-repetition condition. There was no significant difference in age between both groups, *t*(57.86) = −0.99, *p* = 0.32.

#### Materials

The same music clips from Experiment 1 were used in Experiment 2, but this time were presented online.

#### Procedure

The experiment was carried out using videoconferencing software such as Microsoft Teams or Zoom, an online experiment platform called Testable, and Qualtrics. The participants were tested individually. During the study phase, the participant and the experimenter met online through the videoconferencing software. The experimenter sent the participant a web link that directed them to a Qualtrics webpage in which they read the information sheet and expressed their consent.

The participants were randomly assigned to one of two groups. Each group received either one or two repetitions of the 30 target audio clips at a rate of 5 seconds per clip, with a 2-second interval between each clip. The study phase was immediately followed by the instructions of the recognition phase. After the instructions, a set of 20 clips was presented one at a time, followed by a screen with “yes” and “no” buttons, which the participant had to click to indicate whether the music clip was present during the study trials. These 20 clips consisted of 10 targets and 10 nontargets, and as in the study phase, they were presented for 5 seconds, only this time each sound was followed by the buttons “yes” and “no.” Participants were asked to indicate if the tonal sequence at test was present or absent in the studied material by clicking on the “yes” or “no” buttons, respectively. Only once the participant had responded they could hear the next item. The end of the first recognition test was indicated by a screen thanking the participant for their time and reminding them about the next recognition test. For the second and third recognition tests, the participant met the experimenter again through the video conferencing platform in which they received a URL address that took them to the recognition test. They were asked once again to share their screen and to follow the instructions presented. The participants were asked to keep their webcam on during the experiment to ensure their undivided attention. The second test ended with a screen reminding them of the last test to be completed the next day. The third session was identical to the second one, except for the last words on screen which indicated the end of the experiment and thanked the participants for their time.

#### Planned analyses

As in Experiment 1, the rates of hits and false alarms were analysed with generalised mixed-effects models using the Stan modelling language (Carpenter et al., 2017) and the R package *brms* (Bürkner, 2017, 2018) with the default priors. Parameter uncertainty is described by the 95% CI of the posterior distribution in addition to the mean parameter value. The fixed and random effects were the same as in Experiment 1.

Three participants were excluded from the final analyses due to their score at initial test being fewer than five or less correctly identified targets, which we interpret as a lack of compliance with the task. Two of them were in the repetition condition and one in the single presentation condition.

### Results

**Fig. 2:**
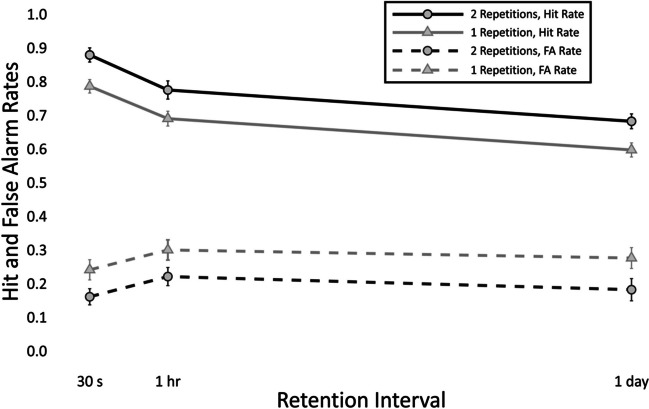
Means and standard errors of hit rates and false-alarm rates at each combination of number of repetitions and retention interval

The means and standard errors of hit rates and false alarm rates are depicted in Fig. 2. The values are reported in the Supplementary Material.

#### Hit rates

There was substantial evidence for an effect of repetition at immediate recall (*b* = −0.78, *SD* = 0.25, CI [−1.28, −0.29]). There was substantial evidence of an effect of delay from immediate to 1 hr (*b* = -0.83, *SD* = 0.25, CI [−1.31, −0.35]), immediate to 24 hr (*b* = −1.35, *SD* = 0.24, CI [−1.82, −0.89]), and 1 hr to 24 hr (*b* = −0.52, *SD* = 0.21, CI [−0.93, −0.11]). There was no evidence of an interaction between delay and repetitions from immediate to 1 hr (*b =* 0.31, *SD =* 0.32, CI [−0.32, 0.93]), from immediate to 24 hr (*b =* 0.36, *SD =* 0.31, CI [−0.25, 0.97]), or from 1 hr to 24 hr (*b =* 0.05, *SD =* 0.28, CI [−0.5, 0.61]).^2^

#### False-alarm rates

There was substantial evidence of an effect of repetition at immediate recall (*b =* 0.69, *SD =* 0.31, CI [0.1, 1.31]). There was substantial evidence of an effect of delay from immediate to 1 hr (*b =* 0.53, *SD =* 0.24, CI [0.06, 1.01]), but no evidence of an effect from immediate to 24 hr (*b =* 0.14, *SD =* 0.26, CI [−0.39, 0.65]), or from 1 hr to 24 hr (*b =* −0.39, *SD =* 0.26, CI [−0.9, 0.11]). There was no evidence of an effect of the interaction between repetition and delay from immediate to 1 hr (*b =* −0.28, *SD =* 0.32, CI [−0.92, 0.34]), immediate to 24 hr (*b =* −0.02, *SD =* 0.34, CI [−0.69, 0.65]), or 1 hr to 24 hr (*b =* 0.26, *SD =* 0.33, CI [−0.38, 0.93]).

We carried out an additional analysis including mode of presentation (in person or online) as a factor. There was no evidence of an effect of mode (*b =* 0.33, *SD =* 0.26, CI [−0.17, 0.84]). There was no evidence for an interaction between mode and delay (*b =* −0.17, *SD =* 0.33, CI [−0.81, 0.49]), between mode and repetition (*b =* 0.40, *SD =* 0.34, CI [−0.63, 0.70]), or amongst mode, repetition, and delay (*b =* 0.17, *SD =* 0.43, CI [−0.67, 1.00]).

This was true also for false-alarm rates. There was no evidence of an effect of mode (*b =* −0.12, *SD =* 0.28, CI [−0.67, 0.43]). There was no evidence of an interaction between mode and delay (*b =* 0.32, *SD =* 0.32, CI [−0.32, 0.93]), between repetition and mode (*b =* 0.02, *SD =* 0.38, CI [−0.74, 0.73]), or amongst repetition, delay, and mode (*b =* −0.31, *SD =* 0.43, CI [−1.16, 0.57]).

### Discussion

Experiment 2, which was carried out online, replicated the findings of Experiment 1. There was no interaction between retention interval and initial degree of learning, confirming that forgetting rates are independent from initial acquisition.

There was no effect of mode, no interactions between mode and number of repetitions, or between mode and retention interval, indicating that the online experiment returned results comparable to those of the in-person experiment. This is relevant given that the move to online testing increased enormously following the COVID-19 pandemic (Peyton et al., 2021), also in studies of forgetting (Baddeley et al., 2019).

The results of the two experiments showed an effect of number of repetitions and forgetting at all delays. Importantly, no interaction between number of repetitions and delay was found, showing that, similarly to verbal material (Rivera-Lares et al., 2022, 2023; Slamecka & McElree, 1983), forgetting rates of nonverbal material are independent from initial degree of learning.

## General discussion

In both experiments, more repetitions increased the initial degree of acquisition, and forgetting occurred with each subsequent test. There was no interaction between initial degree of learning and retention interval, indicating that initial degree of learning does not influence forgetting rates. Forgetting occurred faster during the initial interval, in line with the classic forgetting function of Ebbinghaus (1885/1964).

The results from Experiment 1 were successfully replicated in Experiment 2, indicating that the experiment carried out online yielded similar results to that carried out in person. Taken together, the current results indicate that the rates of forgetting are independent from initial degree of learning (see also Rivera-Lares et al., 2022, 2023; Slamecka & McElree, 1983).

A practical implication of our results is for experiments that compare rates of forgetting between groups that usually do not achieve the same initial degree of learning. Examples of these are older adults relative to younger adults, and clinical populations compared with healthy controls (e.g., Giambra & Arenberg, 1993; Stamate et al., 2020; Walsh et al., 2014; Weston et al., 2018). Frequently, studies in this area assume that the rate of forgetting depends on initial degree of learning (e.g., Craik, 2021; Elliott et al., 2014; Mary et al., 2013; Yang et al., 2016). Accordingly, several studies that compared forgetting curves across groups or individuals used matching procedures to reach a similar learning criterion (e.g., Butler et al., 2007; Fioravanti & Di Cesare, 1992; Muhlert et al., 2010; Nelson et al., 2016; Zerr et al., 2018).

Since the beginning of this debate, matching or not for the level of initial learning yielded opposite outcomes. Huppert and Piercy’s (1979) failed to properly equalize the initial performance of patient HM with that of a group of controls in a test of picture recognition. They claimed that HM showed accelerated forgetting over one week. Freed et al. (1987) contradicted these finding. They assessed the same patient HM also on a forced-choice recognition memory, but to take HM’s initial performance to the same level as that of the controls he was exposed four times to the same set of stimuli, as opposed to the one given to the controls. Their conclusion was that HM did not show faster forgetting.

However, matching performance at encoding, by exposing a group/or an individual to multiple learning trials, is likely to provide differential opportunities for reconsolidation of material at retrieval (Isaac & Mayes, 1999; Jansari et al., 2010; Kopelman & Stanhope, 1997; Zerr et al., 2018).

Our findings are relevant for the study of disorders of memory. If the initial degree of learning does not influence the forgetting rates, studies on group differences in forgetting would not need to equate initial acquisition. Accordingly, in recent years several patients have been reported who show initial learning within the normal range together with rapid forgetting (Baker et al., 2021; Budson et al., 2007; Hart et al., 1988; Hoefeijzers et al., 2013; Zeman et al., 2013). This phenomenon is called accelerated long-term forgetting (De Renzi & Lucchelli, 1993). On the other hand, in the amnesic syndrome or Alzheimer’s disease, the typical finding is an impaired acquisition of new information, accompanied by similar rates of forgetting, and even densely amnesic patients appear to forget acquired information at a broadly equivalent rate to healthy controls (Kopelman, 1985; Stamate et al., 2020; Vallet et al., 2016).

Our results present an interesting challenge for forgetting research. On the one hand, we found that most of the forgetting occurred in the initial interval, in keeping with the classic forgetting curve first described by Ebbinghaus (1885/1964), and which has been replicated with a variety of time intervals, materials, and types (Heller et al., 1991; Murre & Dros, 2015; Wixted & Ebbesen, 1991). Most research about the shape of forgetting curves has attempted to fit a single function (e.g., Fisher & Radvansky et al., 2019; White, 2001; Wixted & Ebbesen, 1991). Slamecka and McElree (1983) found that different degrees of learning led to parallel forgetting functions, which they maintain are difficult to explain using existing theories of forgetting. Wixted (2022) suggests that a power function can result in parallel forgetting, in contrast to other proposed functions. However, even the power function performed poorly in a recent meta-analysis carried out with 916 datasets, aiming to determine the patterns of forgetting (Radvansky, Parra et al., 2022b). Fitting a single function implicitly assumes a single source of forgetting. We suggest that no single forgetting function will produce curves that are both parallel as those shown in the present study and negatively accelerating as in the classic Ebbinghaus’ forgetting curve (see Discussion in Della Sala et al., 2024). Radvansky, Doolen, et al. (2022a) for example propose four separate consecutive forgetting functions operating after different time delays. Such a pattern is, however, currently not strongly supported by other evidence and is likely to be very difficult to test. Our own more cautious suggestion is that two or more underlying processes contribute to the forgetting slopes. We suggest that one of these potential sources of forgetting may be linear and reflect a steady erosion of traces of the memory over time, while another may reflect the classic interference effects (Anderson, 2003). As Wixted (1990) points out, such interference effects are strongly supported by earlier literature (Postman & Underwood, 1973; Underwood, 1957) and can be explained by the fact that the capacity of a cue to aid recollection, is reduced as the number of objects correlated with that cue increases. We suggest that a more cautious approach may be to use the parallel forgetting function as a clue to variables that cause the forgetting curves to diverge.

There is a possibility that testing memory at longer intervals than the ones selected for this study could have produced nonparallel forgetting curves. To our knowledge, no studies on the relationship between rate of forgetting and initial degree of learning have been carried out using auditory nonverbal material at longer intervals.

There is also a possibility that after a certain time, forgetting could stop. Bahrick (1984) tested 773 individuals in the Spanish they learnt at school over a 50-year period and found that forgetting occurred rapidly during the first 3 to 6 years, followed by very minimal change for periods up to 30 years. The memories that could still be recalled after that period, remained accessible for over 50 years, a state that Barick referred to as a “permastore.” Clearly memories for events may be accessible over periods longer than 50 years, but measuring any potential differences in level of retention at such delays is likely to prove difficult.

Most of the forgetting functions explored in forgetting research do not appear to predict or explain our own and similar results (Rivera-Lares et al., 2022, 2023; Slamecka & McElree, 1983). If the same pattern appears consistently across a variety of materials and types of tests, this would suggest the possibility that a general empirical principle of forgetting may have been established (Wixted, 1990). Slamecka and McElree (1983) first identified such a principle with three sets of materials and different types of tests. Recently, we expanded their findings and found the same pattern using different modalities of presentation of verbal material and different delay intervals (Rivera-Lares, 2022), and using different age groups to vary initial degree of learning (Rivera-Lares et al., 2023). In the present study, we found the same pattern using nonverbal auditory material. This evidence indicates that forgetting rates do not depend on the initial degree of learning and suggests that forgetting is a process independent from learning.

## Supplementary Information

Below is the link to the electronic supplementary material.

Supplementary file1 (DOCX 18 KB)

## Data Availability

The datasets generated and analyzed during the current study, as well as the materials used are available in the OSF repository (https://osf.io/g58qh/).

## Footnotes

1 The results were confirmed by more traditional analyses. A repeated-measures ANOVA found no interaction between delay and number of repetitions, *F*(2, 318) = 0.452, *p* = .64, for rates of hits or for rates of false alarms, *F*(2, 156) = 1.750, *p* = .18. Moreover, we analysed *d*-prime scores, and no interaction was found from immediate to 1 hr (*b* = −0.15, *SD* = 0.2, CI [−0.55, 0.25]), from immediate to 24 hr (*b* = −0.16, *SD* = −0.16, CI [−0.57, 0.25]), or from 1 hr to 24 hr (*b* = −0.01, *SD* = 0.2, CI [−0.4, 0.38]).

2 The outcome was confirmed by more traditional analyses. A repeated-measures ANOVA showed no interaction between delay and number of repetitions for hit, *F*(2, 336) = 0.021, *p* = .98, or for false-alarm rates, *F*(2, 165) = 0.038, *p* = .96. The *d*-prime analysis showed no interaction from immediate to 1 hr (*b* = −0.14, *SD* = 0.19, CI [0.24, −0.51]), from immediate to 24 hr (*b* = −0.1, *SD* = 0.18, CI [0.26, −0.47]), or from 1 hr to 24 hr (*b* = −0.04, *SD* = 0.19, CI [0.4, −0.38]).

## Code availability

The R code is available in the OSF repository (https://osf.io/g58qh/).
