# Supplementary material for: Influence of degree of learning on rate of forgetting of tonal sequences
Source: Mem Cognit. 2024 Jul 17;53(2):682–91. doi: 10.3758/s13421-024-01597-6 (PMC11868304; doi:10.3758/s13421-024-01597-6)
Supplement: Supplementary file 1 — Supplementary file1 (DOCX 18 KB) [file 13421_2024_1597_MOESM1_ESM.docx]

**Table S1**

Means and standard errors of hit and false alarm rates at each combination of number of repetitions and retention interval in Experiment 1.

|  | Hits | | | | | |
| --- | --- | --- | --- | --- | --- | --- |
| Repetitions | Immediate | | 1 hour | | 1 day | |
|  | *M* | *SE* | *M* | *SE* | *M* | *SE* |
| Two | .84 | .02 | .75 | .03 | .67 | .03 |
| One | .72 | .02 | .61 | .03 | .56 | .03 |
|  | False Alarms | | | | | |
| Two | .17 | .02 | .19 | .02 | .14 | .03 |
| One | .26 | .03 | .31 | .03 | .32 | .03 |

**Table S2**

Means and standard errors of hit and false alarm rates at each combination of number of repetitions and retention interval in Experiment 2.

|  | Hits | | | | | |
| --- | --- | --- | --- | --- | --- | --- |
| Repetitions | Immediate | | 1 hour | | 1 day | |
|  | *M* | *SE* | *M* | *SE* | *M* | *SE* |
| Two | .88 | .02 | .78 | .03 | .68 | .02 |
| One | .79 | .02 | .69 | .02 | .60 | .02 |
|  | False Alarms | | | | | |
| Two | .16 | .02 | .22 | .03 | .18 | .03 |
| One | .24 | .03 | .30 | .03 | .28 | .03 |

**Table S3**

Means and standard errors of hit and false alarm rates at each combination of number of repetitions and retention interval in the analysis of the combined data from Experiments 1 and 2.

|  | Hits | | | | | |
| --- | --- | --- | --- | --- | --- | --- |
| Repetitions | Immediate | | 1 hour | | 1 day | |
|  | *M* | *SE* | *M* | *SE* | *M* | *SE* |
| Two | .86 | .02 | .76 | .02 | .68 | .02 |
| One | .76 | .02 | .65 | .02 | .58 | .02 |
|  | False Alarms | | | | | |
| Two | .17 | .02 | .21 | .02 | .16 | .02 |
| One | .25 | .02 | .31 | .02 | .30 | .02 |
